# The Welfare of Cattle in Different Housing Systems

**DOI:** 10.3390/ani15131972

**Published:** 2025-07-04

**Authors:** Bogumiła Pilarczyk, Renata Pilarczyk, Małgorzata Bąkowska, Agnieszka Tomza-Marciniak, Beata Seremak, Ewa Kwita, Marta Juszczak-Czasnojć, Paulius Matusevičius, Ramutė Mišeikienė

**Affiliations:** 1 Department of Animal Reproduction Biotechnology and Environmental Hygiene, Faculty of Biotechnology and Animal Husbandry, West Pomeranian University of Technology in Szczecin, Klemensa Janickiego 29, 71-270 Szczecin, Poland; bogumila.pilarczyk@zut.edu.pl (B.P.); agnieszka.tomza-marciniak@zut.edu.pl (A.T.-M.); beata.seremak@zut.edu.pl (B.S.); ewa.kwita@zut.edu.pl (E.K.); marta.juszczak-czasnojc@zut.edu.pl (M.J.-C.); 2 Laboratory of Biostatistics, Bioinformatics and Animal Research, Faculty of Biotechnology and Animal Husbandry, West Pomeranian University of Technology in Szczecin, Klemensa Janickiego 29, 71-270 Szczecin, Poland; renata.pilarczyk@zut.edu.pl; 3 Department of Animal Nutrition, Veterinary Academy, Lithuanian University of Health Sciences, Tilžės 18, LT-47181 Kaunas, Lithuania; paulius.matusevicius@lsmu.lt; 4 Institute of Animal Rearing Technologies, Veterinary Academy, Lithuanian University of Health Sciences, Tilžės 18, LT-47181 Kaunas, Lithuania; ramute.miseikiene@lsmu.lt

**Keywords:** cattle, environmental enrichment, rearing system, stereotypies, welfare

## Abstract

The review provides an overview of research concerning the assessment of cattle welfare in different housing systems. Hence, it restricts its scope to factors known to have a particular influence on the expression of their natural behaviours. It analyses the impact of housing systems on social and maternal bonds, as well as on the health and productivity of animals and on the feeding behaviour and physical activity of animals. It also pays attention to the occurrence of stereotypies, indicating the quality of the environment in which animals live, and attempts to determine the extent to which environmental enrichment improves welfare. It can be seen that welfare can vary significantly depending on the cattle rearing system. In intensive rearing environments, weaning calves and limited space often result in stress and behavioural disorders (e.g., cross-sucking). Extensive systems, offering access to pasture and longer cow–calf contact, usually provide higher levels of welfare. A freestall system allows greater freedom of movement and social contact but requires appropriate management to prevent aggression; in contrast, the tethering system limits movement, which increases the risk of stress and health problems. It has also been shown that enriching the living space of animals can significantly improve their welfare, regardless of the housing system. By balancing productivity with ensuring that the cattle are able to express their natural behaviours and maintain good health, it is possible to benefit both the animals and the agricultural sector as a whole, increasing its profitability and gaining consumer confidence.

## Simple Summary

Recent years have seen greater consumer interest in animal welfare, resulting in more responsible purchasing of animal products and the implementation of more sustainable farming practices. In the European Union, improving animal welfare is a priority, supported by both scientific research and public expectations. The European Green Deal considers animal welfare as a key element of sustainable development, encompassing the physical and mental health of animals. Among cattle, welfare levels are influenced by various factors, including housing conditions, living space, and social interactions. The main welfare challenges for cattle include stress, stereotypies, health problems (e.g., lameness, mastitis, and metabolic disorders), and disruption of the herd hierarchy, leading to aggression in unstable groups. Well-being can also be improved by environmental enrichments, which have been found to reduce stress and promote natural behaviour.

## 1. Introduction

Animal welfare is a complex concept encompassing both the physical and mental health of an animal and its ability to express its natural behaviour in its environment. It entails not only the absence of disease and injury but also the provision of appropriate conditions that allow the animal to reach its full potential [1,2,3]. Therefore, to assess the welfare of an animal, it is necessary to consider objective indicators, such as health and behaviour, and the subjective sensations of the animal [4,5].

Of the many factors that influence animal welfare, one of the most significant is the quality of the husbandry environment; this can be determined by, inter alia, the quality and availability of resting places, the area per animal, and the possibility of using paddocks or pastures. For cattle, it is important to optimise their housing conditions, the provision of their stands, and production technologies to ensure adequate welfare.

One of the most important tools for assessing animal welfare is monitoring animal behaviour. Regular observation allows the identification of negative factors and the introduction of any remedial measures. Again, any resulting changes benefit not only the animals but also the entire agricultural sector, increasing its profitability and improving consumer confidence.

The members of the European Union in particular place an emphasis on improving the living conditions of animals. In response to scientific observations and the growing awareness of citizens, the EU has passed legislation imposing increasingly stringent requirements on animal breeders. Animal welfare has been included in the European Green Deal strategy, which emphasises the importance of this issue as a key element of sustainable development.

Recent years have witnessed a growth in consumer awareness of animal welfare. This translates into more informed choices. Consumers are increasingly willing to pay a premium price for products from animals raised in conditions prioritising their welfare. This attitude has a direct impact on the market and is forcing producers to introduce more ethical and environmentally friendly farming practices.

However, it is possible to meet the natural behavioural needs of livestock by implementing modern ethical husbandry practices that exceed current standards. These practices reduce stress, aggression levels, and the risk of self-harm, while improving comfort. Such changes result in a higher quality of life for the animals, which translates into improved production efficiency and better quality products for the farmer [6].

Animal welfare assessment requires the consideration of a number of variables. As such, it is necessary to employ a comprehensive approach to welfare assessment that can include these factors [7].

Regardless of the type of housing system, the welfare of cattle depends on many factors, including environmental conditions, their social interactions, and the possibility of realising natural behaviours.

Cows kept on a tether are more likely to have welfare disorders than those in a freestall system. This is due to the specific nature of the system, with the main threats to welfare being too short a tether and too small a stall. In turn, a freestall system may suffer from inadequate amounts of bedding and poor hygiene, which can result in lameness and mastitis. Lack of access to pasture also plays a significant role in reducing animal welfare [8].

The aim of this work is to review the available literature on the impact of housing conditions on the well-being of cattle, with a particular emphasis on the possibility of expressing natural behaviours, productivity, and health. It discusses the most important factors known to influence the assessment of cattle welfare. It focuses on the impact of freedom of movement, access to water, and animal hygiene on well-being. It also examines the bond between mother and calf, psychological comfort, and health control, which also influence the maintenance of animal welfare, and examines factors that promote and prevent the occurrence of behavioural and health disorders.

The corpus for the review was acquired by searching PubMed, Web of Science, Google Scholar and Scopus using the following keywords: cattle, welfare, housing systems, dairy cows, beef cows, cattle housing, environmental enrichment, stereotypies, behavioural disorders of cattle, cattle feeding, calves, cattle behaviour, extensive housing system, intensive housing system. All information was derived from experimental, original, and review articles, as well as books and reports.

The search of the PubMed, Web of Science, Google Scholar, and Scopus databases yielded 1450 original results. Google Scholar returned more results, including less relevant ones, such as theses or preprints. During the following selection process, 410 duplicates were removed, followed by another 500 that were unrelated to the topic, leaving 540 publications. Of these, 85 were not available in full text, and 80 did not meet the substantive criteria, ultimately qualifying 375 publications for analysis. From this group, 127 of the most relevant works were selected for detailed discussion.

The final articles were selected based on the following inclusion criteria:

- The article concerned the welfare of cattle in a variety of housing systems, including extensive, intensive, freestall, and tethering systems.
- The article detailed the impact of the housing system on the behaviour, health, and performance of the animals, taking into account factors such as living space, social interactions, access to pasture, and nutritional conditions.
- The article included an analysis of stereotyped behaviour in cattle and methods of environmental enrichment to improve welfare.
- The article presented the results of research on the impact of the housing system on the relationship between mothers and calves and the structure of the herd hierarchy.

The information was then analysed, categorised, and presented in sections to effectively cover the scope of the review.

## 2. Welfare of Cattle in Different Housing Systems

In all housing systems, cattle welfare depends on many variables, with most concerning environmental conditions and the social interactions between the animals. However, significant variation exists in between types of cattle housing, and these have considerable effects on the behaviour, health, and welfare of animals (Table 1). The most favourable conditions for maintaining behavioural stability and welfare were noted in cattle living in the wild. These groups maintain lasting mother–calf bonds and are characterised by a complex age structure and numerous diverse interactions. They have constant access to pasture, which allows for the implementation of natural activities such as grazing, resting, or creating relationships in the herd. Stereotypic behaviours are not noted in such herds, disease is rare, stress levels are low, and environmental conditions are as close to natural as possible. The situation is slightly different for cattle kept in extensive and intensive systems. Despite greater oversight by farmers, the mother–calf bond is weaker, and the herd is more likely to demonstrate stereotypic behaviours, which are reflected in lower welfare; it is also at a higher risk of disease and stress, which is attributed to limited space, higher stocking density, and insufficient access to pasture.

Dairy cattle are generally kept in conditions as far from natural as possible, with the calves being separated from their mothers immediately after birth and the cows being kept in closed housing with high stocking densities (Table 1).

### 2.1. The Importance of Social and Maternal Bonds

A very important element affecting animal welfare is the structure of the bond between mothers and calves, as this plays an important role in shaping the social and emotional behaviour of cattle [9]. In the case of wild cattle, these bonds are particularly strong: mothers maintain close relationships with their adult daughters, which support matrilineal structures in the herd and serve as the basis for harmonious social functioning (Table 1). The calves also form strong relationships with other individuals, which foster feelings of security and support the social behaviours characteristic of cattle: indeed, cattle originally lived in herds with strong social bonds [9].

#### 2.1.1. Separation of Calves

Sudden separation of cows from their calves is known to evoke mild stress for both parties, manifested by increased heart rate and increased vocalisation within the first 10 min, as well as elevated cortisol levels persisting for six to eight hours after separation [10]. Such situations are characteristic of artificial calf rearing systems, which are based on individual pens and involve limited contact between cow and calf. Early weaning of calves from their mothers often leads to undesirable behaviour, known as stereotypic behaviour or stereotypies, such as foreskin sucking, tongue rolling, excessive licking, biting and tube sucking, as well as excessive self-feeding [11,12]. The type of stereotypies observed in calves is closely related to their housing conditions. In individual pens and kennels, the animals most often show self-aggressive stereotypies such as sucking, licking and biting objects, playing with their tongues, and head bouncing. In group pens, where animals are in contact with each other, social stereotypies are also observed, involving mutual sucking and manipulation of other individuals. The intensity of these behaviours increases especially during the feeding period [13].

Therefore, to ensure their optimal social development, it is recommended that calves are separated from their mother no earlier than 24 h after birth. Conversely, it should be borne in mind that prolonged residence with the mother can encourage the formation of strong emotional bonds that prevent the calf from establishing relationships with other animals [14,15]. Furthermore, raising calves in small closed groups limits their ability to form lasting social bonds, and such practices disrupt the natural social structures in the herd.

#### 2.1.2. Extensive Housing System

In the extensive beef cattle system, a strong bond is observed between calves and their mothers for the first few months of their lives, which becomes less intense between mothers and adult daughters (Table 1).

In addition, cow and calf are less able to interact frequently, e.g., by licking and rubbing one another, aimed at meeting their natural needs. The presence of the mothers also allows the calves to satisfy their natural need to suckle, which translates into a lower incidence of undesirable behaviour such as suckling other calves (cross-suckling) [16]; further, calves are able to control the frequency and timing of their feeding, thus replicating natural conditions more closely.

In extensive herds, however, natural social bonds are more limited. This is especially apparent in technological groups where animals vary considerably in age: while these groups allow some interaction, the regular rotation of animals results in fewer bonds forming between herd members (Table 1). As a result, the conditions do not support the development of complex social bonds, and long-term social bonds are rare.

#### 2.1.3. Intensive Housing System

In intensive beef and dairy farming systems, calves are separated from their mothers shortly after birth, which inhibits the development of maternal bonds and disrupts natural social behaviour [17]. Under natural conditions, the bond between mother and calf forms immediately after birth and usually lasts for at least one year [18]; however, in an intensive system, without this bond, calves are more likely to develop stereotypic behaviours, especially just after weaning (Table 1). In addition, restricted suckling intervals, too little milk, and lack of natural suckling opportunities lead to the development of behaviours, such as cross-suckling, which can persist throughout life [19].

Cattle need to satisfy their behavioural needs by maintaining social relationships with other individuals, such as the bond between mother and calf: such bonds have been found to reduce the chance of undesirable behaviours and stress. It is therefore important to pay attention to calf behaviour, as it can be an early indicator of poor welfare and allow action to be taken to reduce stress. It is also necessary to be aware of the limitations of intensive cattle management systems.

### 2.2. Comparison of Intensive Housing Systems

The two main systems used in dairy farming are freestall housing and tether housing. These differ with regard to, inter alia, access to water, stall hygiene, and freedom of movement. As such, the choice of housing will have a considerable impact on animal welfare.

#### 2.2.1. Restrictions and Freedom of Movement

In the freestall system, the cows are provided free access to the resting, feeding, and milking areas of the barn, which encourages natural behaviours such as movement, rest, and socialisation. These systems allow for greater expression of animal social behaviour but also require appropriate herd management to avoid aggression, especially in large stocking densities [20,21]. Studies indicate that implementing a freestall system lowers the incidence of udder disorders. Teat injuries were observed less frequently in the freestall system (0.2%) than in the tethered housing system (0–1.6%) [22]. Moreover, the freestall system reduces the risk of ketosis (RR = 1.59, *p* < 0.01) and improves the fertility status index (82.8 vs. 67.3%, *p* < 0.01) [23], although it may also increase the likelihood of lameness, especially as a result of greater cow mobility [24]. On the other hand, the tethering system restricts freedom of movement, which increases the risk of injury and stress (Table 1). However, it allows for better control of the cows and reduces the degree of competition for feed. While this is an advantage for smaller herds, the reduction in freedom negatively affects cow welfare, and the system is gradually losing popularity [21].

#### 2.2.2. Animal Hygiene

An important element in assessing cow welfare is hygiene. Cows kept in tethered systems are more likely to have dirty sides and upper parts of the limbs than those in freestall systems (43% vs. 25%), while those in loose-stall systems are more likely to have dirty lower parts of the limbs than those in tethered systems (51% vs. 33%) [21]. Tethered systems are also characterised by a higher percentage of cows with dirty udders, indicating hygiene problems [25]. A study of the tie stall system found 86.67% of cows to have skin lesions, indicating that the environment had a negative impact on skin condition [26]. Lack of adequate hygiene increases the risk of udder inflammation and worsens health conditions, including an increased incidence of lameness [27,28]. However, both systems are associated with a risk of lameness, which is rooted in the restricted movement of the cows and the environmental conditions.

#### 2.2.3. Mobility and Social Interaction

The two systems also differ with regard to mobility and social interactions (Table 1). Cows in the free-range system have more freedom of movement, which encourages social interactions such as head bumps; they also engage in movement twice as frequently as in the tethering system [29]. Although the free-ranging system allows for natural behaviours such as movement and social interactions, these same behaviours can lead to greater health risks, especially in feeding and watering areas, where competition often occurs [30]. The tethering system is also beset by limited space and inappropriately designed stalls such as being too narrow or too short or with bedding obstacles; such conditions increase stress by providing less comfort and making it difficult for the animals to rest [31].

#### 2.2.4. Access to Water

Regarding access to water, the freestall system offers better access to higher quality water from drinkers. In the tethered system, water is often distributed to a large number of cows through a trough system, which can result in inadequate water supply; in addition, access to water is sometimes limited, and the drinkers are less frequently cleaned and inspected, resulting in lower water quality. Restrictions on access to water in a tethering system can also increase aggression in the herd. In contrast, in the freestall system, cows have free access to drinkers, which are regularly cleaned and maintained, ensuring better hygiene and availability of fresh water. This in turn translates into higher milk yields and welfare [32].

#### 2.2.5. Psychological Well-Being

Noticeable differences in the psychological well-being of cows have also been reported between the two systems. The freestall system has been found to demonstrate a higher welfare index, as measured by the Quality of Behaviour Assessment (QBA) method, indicating greater psychological comfort [25]. Cows raised in this system can express their natural behaviour, which translates into a better emotional state. In contrast, in a tethering system, the animals have limited space and cannot easily meet their natural needs; while they can have more frequent contact with the keeper, such conditions can lead to stress, fear of people, and a deterioration in the performance of cows that show signs of anxiety [33].

#### 2.2.6. Monitoring Health

Due to the greater possibility for movement in the freestall system, it is also easier to monitor animal health. In contrast, the conditions in the tethered system, i.e., the lack of space to move freely, promote an increased risk of disease, reduce the effectiveness of intervention, and complicate the response to health problems [34].

Based on these aspects of animal husbandry, the freestall system seems to be more beneficial for cattle breeding with regard to ensuring animal welfare, the possibility of expressing natural behaviours, and reducing the occurrence of stereotypies.

### 2.3. Extensive Housing System—Eating Behaviour and Physical Activity

When fed by their mother, calves suckle for 8–11 min per feeding and the frequency of suckling decreases with age. Such feeding not only plays a nutritional role but also stimulates the development of the digestive system and satisfies the instinctive need to suckle [14]. When calves cannot be fed by their mothers, it is essential to provide them with milk or milk replacement, preferably at frequent intervals, to support their physiological and psychological development. Such formulations should contain adequate protein (25–28%) and fat (15–17%). Calves should be able to suckle as naturally as possible to prevent health problems such as choking or excessive neck muscle tension [35].

#### 2.3.1. Grazing

On pasture, cows typically feed for between six and twelve hours per day, while this time is limited to about four hours in enclosed systems, such as barns [36,37]. The dominant behaviours on pasture are grazing, ruminating, and resting, which together account for 90–95% of the day [38]; in addition, most of the grazing takes place during the day, while the night consists mainly of resting and rumination. Cows decide for themselves where and when they will graze. They usually prefer to take their feed (fresh grass) in cooler weather, but they will adapt to the current weather [39]. Grazing on large pastures, which provide space for the exercise of natural social instincts, improves animal welfare and the physical health of the cows and increases the quality of their milk, which contains higher levels of beneficial fatty acids. Such grazing also reduces stress and fosters stronger social bonds, thus lowering the risk of conflict and aggression (Table 1) [40].

O’Connell et al. [41] and Miller and Wood-Gush [42] report significantly lower intensity of agonistic interactions in pasture compared to confined environments. In indoor housing systems, two distinct peaks of aggression were observed, which correlated with the timing of feed administration. The vast majority of aggressive behaviour was also observed in the feeding zones, indicating that competition is particularly high in these areas; indeed, in barns, cows may have limited access to the feed table, leading to higher levels of stress, especially among animals that are lower in the hierarchy. Furthermore, without pasture, the animals do not have adequate space to fulfil their natural needs, leading to frustration and stress (Table 1). In contrast, in an extensive system, animals have access to pasture for most of the year, thus improving their welfare [39,40].

#### 2.3.2. Rest

Olmos et al. [43] report that cows in pasture spent more time lying down than those in barns (42.7% vs. 37.7%) and had longer lying periods (50.3 min vs. 39.3 min). Cows in barns stand for longer in stalls, which may indicate less comfort and a greater risk of health problems such as hoof disease; they are also more likely to demonstrate a loss of synchronisation when lying down with other cows, i.e., fewer than 45% of cows lying down at the same time compared to 90% in pasture, suggesting a lower level of welfare [42]. Lying down is an important indicator of the welfare of dairy cows, as it reflects their physiological and behavioural needs. Dairy cows typically spend about 10–14 h per day lying down [44], and deviations from this norm may indicate health problems, stress, or inadequate housing conditions [45,46]. Both excessively short and excessively long periods spent lying down may indicate the presence of health problems or discomfort, signalling the need for intervention (Table 1).

#### 2.3.3. Physical Activity

A study by Dohme-Meier et al. [47] showed that on average, cows on pasture took 3390 steps per day, indicating a natural level of activity for this species; in contrast, they took an average of only 748 steps per day in the tethering system, the most restricted in terms of locomotion [48]. Again, the physical activity level of cows can be considered a measure of welfare, as it reflects the extent to which animals can exhibit their natural behaviour [49]. Cows that were allowed to go out for a short walk were quicker to lie down and get up than those that stayed in the barn all the time [50].

#### 2.3.4. Social Relationships

The lack of space and the numerous groupings of animals kept in intensive systems have the effect of simplifying the social bonds within the herd (Table 1). While the extensive system may have weaker bonds than wild groups, they nevertheless promote social stability, which improves animal welfare [14,17].

When kept in an extensive system, cattle usually have access to an appropriate space within which they can express their natural needs and behaviours. In cases where the frequency and length of feeding are not restricted, young animals have been found to exhibit correct development of the digestive system; they also form deeper bonds with the mother and demonstrate better interactions with other individuals.

### 2.4. Impact of the Housing System on Animal Health and Performance

#### 2.4.1. Bedding—Types

A very important element in cow welfare is the choice of area and the quality and quantity of bedding used. Traditionally, straw has been used for bedding in livestock housing, providing a warm and soft substrate. However, in recent years, livestock farmers have increasingly warned of its potential to encourage bacterial growth and thus a higher risk of infections such as mastitis [51]. In response to these concerns, many farmers have switched to alternative materials such as wood shavings, sawdust, or sand, which can be more hygienic and reduce the risk of infection [52,53]. Although these materials are not as warm as straw, they can provide a solution for farms where the availability and cost of straw may present a problem. Cows prefer soft and well-insulating surfaces, such as a deep layer of straw or rubber mats; when provided, their use results in longer lying time, which is important for health [54,55]. Indeed, cows kept on straw bedding were found to enjoy longer total lying times during the day than those on sand (749 ± 16 vs. 678 ± 19 min.) [54]. Soft surfaces reduce discomfort and improve cow welfare, while hard surfaces can cause health problems and limit rest.

A lack of adequate bedding, especially in freestall systems, has negative health consequences due to an increased risk of injury, including udder damage, as well as reduced milk yield and shorter life expectancy. The lack of a comfortable resting area can also increase the risk of leg injuries and skin lesions (e.g., abrasions) [56,57,58]. In addition, hard surfaces such as concrete increase the incidence of injuries when standing up and lying down, particularly in the knees, leading to swelling [59].

#### 2.4.2. Alternative Bedding Material

Rubber mats or mattresses filled with synthetic material can be used as alternatives to traditional bedding materials and can reduce pressure on the joints and improve the comfort of resting animals. Cows on mats spend more time lying down (approx. 9.37 h/d) than those on concrete (8.13 h per day), which indicates greater comfort [59]. Rubber mats have been shown to have a positive effect on cow comfort and to have a positive impact on cow welfare by providing assistance when lying down and standing up [59]. However, the use of mats has been associated with an increased incidence of ankle joint injuries [8,58,60].

The use of rubber mats can be particularly beneficial in conditions where the amount of bedding is limited, providing the animals with adequate resting comfort while reducing costs [59].

## 3. Main Welfare Issues for Cattle

The key problems in maintaining cattle welfare stem from the drive to intensify production, which often prioritises efficiency at the expense of animal health and comfort. While these problems vary according to the goal of production, beef and dairy cattle farming have many in common. Among beef cattle, the key problem associated with intensive farming is limited space in barns, where high stocking densities and lack of adequate ventilation lead to heat stress; in addition, poor feeding increases the risk of numerous health problems, such as acidosis, lameness, or ketosis. A clear correlation has been noted between animal welfare and stereotypic behaviour, the latter of which represents an important indicator of the quality of the environment in which cattle are housed.

### 3.1. Stocking Density

Regardless of the housing system, excessive stocking densities disrupt their natural social behaviours, such as forming stable groups and establishing hierarchies within the herd (Table 1). Under such conditions, the frequency of conflict and aggression between animals increases, leading to numerous physical and psychological injuries. The increased rivalry and difficulties associated with adapting to the confined space place increased stress on the cows, deteriorating their behavioural health and impairing their physical condition and biological functions. Long-term stress associated with overcrowding increases the risk of disease, reduces productivity, and decreases the quality of life of the animals.

### 3.2. Lack of Access to Pasture

Among dairy cattle, intensive management entails a lack of access to pasture, which prevents the cows from expressing their natural behaviour (Table 1). Grazing on pasture almost completely eliminates the occurrence of stereotypies in cattle, which return after re-tethering [61,62]. In addition, moving cows from tethered to freestall barns was found to significantly reduce the frequency of stereotypic behaviour [62]. Studies have found access to pasture to be associated with reduced oral stereotyped behaviours among dairy cattle that had previously been tethered [63].

### 3.3. Improper Nutrition

The occurrence of behavioural disorders has also been found to be associated with inappropriate amounts of feed, infrequent feeding, and using feed with an inappropriate composition. Providing large amounts of concentrated feed with small amounts of roughage leads to a reduction in the feed intake time and filling of the digestive tract [64]; such practices contradict the natural feeding pattern of cows, i.e., in which they consume larger amounts of feed with a lower feed value [65]: under natural conditions, cattle spend much of their active time foraging and chewing [66], and not being allowed to engage in these behaviours can result in feelings of hunger and frustration. Excessive concentrated feeds and a deficiency of fibre in the diet reduce rumination time; this has been associated with disturbances in the rumen microflora and discomfort, resulting in the development of unnatural oral behaviour, such as object sucking or pseudo-feeding [67,68,69]. Such behaviours can also result from unmet instincts and more serious health problems, including metabolic disorders [68]. Indeed, greater numbers of stereotypic behaviours have also been observed when food is restricted compared to an ad libitum system [70]. Again, this relationship is explained by the frustration associated with not being able to fully satisfy food needs, which is particularly common in cattle tethering systems [62].

Stereotypic behaviour can also be perpetuated by nutritional deficiencies, such as a lack of sodium chloride; in such cases, the cow may lick the pen or random objects [71]. Studies indicate that stereotypies in cattle increase in the first two to four hours after feeding [61].

In an extensive system, where cows have greater access to pasture and natural feed, animals are more likely to follow their instincts, and less likely to display abnormal behaviour.

### 3.4. Illness

Animals kept in tethering systems often suffer from mastitis and metabolic diseases, such as ketosis and acidosis (Table 1), especially in the postpartum period, as well as lameness, as a result of intensive use and inappropriate housing conditions [21,72,73].

Regula et al. [22] report a significantly greater frequency of lameness in cattle that rarely had access to open areas compared to those that used them regularly (19% vs. 12%). The risk of lameness was about 30% lower in the freestall system with access to runs than in systems with minimal access. In addition, cows in the tethered system (8–21%) more commonly presented with skin injuries around the hock than those in the loose-stall system (3–8%). Calluses on the carpal joints were also more frequent in the tethered system (58–65%) than in the freestall system (4–17%).

Keeping dairy and beef cows and their calves in an intensive indoor system presents many challenges for the farmer, as the environment limits their ability to perform their natural behaviours, and the cows are forced to live in unnatural social groups [74,75].

### 3.5. Calving

Some mating practices, such as mating with a bull with a large muscle mass (e.g., Belgian Blue, Blonde d’Aquitaine, Charolaise, or Piemontese), can result in the conception of an oversized calf. Where the pelvis is too narrow, the cow may experience difficulty in giving birth. This can pose a serious risk to both cow and calf, resulting in very high levels of stress and pain to the mother and often leading to the death of the calf. This risk is particularly high in primiparous cows and in purebred herds, where both maternal and paternal genes increase the chance of giving birth to a large calf. In order to reduce the risk of difficult births, care should be taken to ensure that the cows are in good condition before delivery and to be prepared to carry out a caesarean section in the event of complications.

### 3.6. Socialisation

The social structure of a herd may also be disrupted when it is too large. Fraser and Broom [76] estimate the social memory capacity of cattle to be around 50–70 individuals, a number frequently exceeded in large dairy herds. Although the literature on this subject is relatively sparse, there is a consensus that frequent changes in herd composition and keeping animals in very large groups are stressors and can lead to increased levels of aggression. However, it is worth emphasising that the severity of these negative effects depends on a number of factors, including the herd management practices used.

Cattle, regardless of age, have a natural need to explore their surroundings. This behaviour, called exploration, is innate and allows animals to function effectively in their environment.

## 4. The Effect of Breed on Cattle Welfare

It is believed that cows feel most comfortable when they are provided with care and proper nutrition and environmental conditions that allow them to express their natural behavioural patterns. However, it should be remembered that the housing conditions appropriate for one breed may not be suitable for others: one breed may feel comfortable while the other could experience lower well-being. A number of disorders may manifest themselves as stereotypic behaviours or stereotypies.

The incidence of stereotypies such as tongue rolling in dairy cows varies considerably by breed. One study conducted on a large farm in the United States found 29.0% of all cows to exhibit this behaviour at least once during three consecutive lactations; in addition, Jersey cows were twice as likely to exhibit tongue rolling (33.4%) as Jersey–Holstein crosses (17.2%) [77]. Analysing the influence of lactation and breed, tongue rolling was noted in 21.9% of primiparous Jersey cows compared to 14.8% of primiparous Jersey–Holstein cows. This difference was even more pronounced in multiparous cows (i.e., those in their second or subsequent lactations), reaching 40.0% in Jersey cows compared to 22.1% in Jersey–Holstein crosses [77]. Other authors have reported a significantly higher incidence of tongue rolling in Swedish Red and White cows, with one study noting 42% of individuals [62] and another 73% [70]. In addition, primiparous Jersey cows exhibited a greater probability of tongue rolling with increasing days in lactation, while primiparous Jersey–Holstein cows did not. A similar trend was also observed in multiparous Jersey cows, with only a small change in Jersey–Holstein crosses [77]. This type of stereotypy has also been noted in calves, although [78] it appears to be relatively rare in Fleckvieh calves and much more common in Jersey calves [79]. Tongue rolling was found to be significantly more common in Swedish Red and White calves [70].

Some authors have noted a greater predisposition to cross-sucking in Jersey calves than in Danish Red or Holstein [79]; however, no such predisposition was observed in Holstein, Brown Swiss, or Simmental cows [80]. Studies on the Fleckvieh breed [78] suggest that genetic variation between bulls may play a role, with the percentage of offspring displaying this behaviour ranging from about 2.1% to over 16%. Not all breeds display the same tendency to stereotypy, which is reflected in varying percentages of behaviours such as tongue rolling or mutual suckling. In addition, Keil et al. [80] showed that the presence of such behaviour in heifers within a herd is the best indicator of the later occurrence of cross-sucking in cows; this suggests that cross-sucking in cows may represent a continuation of behaviours formed at the rearing stage, and that effective prevention requires action to be taken as early as possible, while the animals are still young.

These results suggest that the chance of stereotypy is influenced by both genetic and environmental factors. However, some breeds appear to be more predisposed to certain stereotypies, highlighting the importance of considering breed when assessing animal welfare and improving welfare conditions [77].

## 5. Approaches to Enriching the Environment

One effective strategy that can improve animal welfare is environmental enrichment i.e., modifying their environment to meet their natural behavioural needs. Such activities have been found to reduce stress, reduce undesirable behaviours (e.g., stereotypies or aggression), and improve the physical and mental health of the animals. As indicated by Boissy et al. [81], an enriched environment is designed to meet the natural behavioural needs of animals, thus contributing to reducing stress and improving overall well-being, understood as both the absence of suffering and the presence of positive emotions.

Bloomsmith et al. [82] distinguish five categories of environmental enrichment: social, occupational, physical, sensory, and nutritional (Table 2). Each category influences animal welfare by providing appropriate stimuli and enabling natural behaviours, but in different ways. Providing social enrichment by increasing contact with other individuals will not only reduce the stress but also stimulate social behaviour. In turn, modifying the animal’s environment by using additional elements or changing the size of the enclosure can satisfy the need for physical activity and exploration of the environment. Occupational enrichment can satisfy the need for cognitive abilities and reduce boredom. In turn, sensory enrichment, through the use of olfactory or auditory stimuli, will satisfy the need to stimulate individual sensory organs (Table 2).

Among bovines, environmental enrichment includes diverse activities aimed at adapting their living conditions to their natural needs, resulting in improved comfort and welfare. According to Broom [83], designing such an environment for cows requires taking into account their complex cognitive abilities, which have a significant impact on their behaviour and well-being.

However, while these measures improve the comfort and health of the animals and reduce undesirable behaviour, some problems can arise following the introduction of new elements into the environment.

### 5.1. Physical Space

A fundamental aspect of environmental enrichment is the physical modification of space. For example, dividing the barn into functional zones has been found to reduce aggression between animals and allow weaker individuals to shelter: the introduction of wooden walls in calf pens reduces the number of aggressive interactions between animals (*p* < 0.01), allowing weaker individuals to shelter from dominant ones [84,85]. In turn, increasing the available space results in a marked increase in the playful activity of the calves (119 vs. 67 s/24 h), as confirmed by Jensen et al. [86], and the addition of stimuli such as fresh bedding, especially during morning feeding, further stimulates this activity [87].

Providing a comfortable environment for rest and isolation is particularly important for adult cattle. Research by Proudfoot et al. [88,89] show that cows prefer to use secluded areas during parturition or illness; such measures reduce their stress levels by allowing them to satisfy their natural need for seclusion. The physical comfort of the animals also depends on the quality of the lying area. As indicated by Tuyttens [90] and Norring et al. [91], the use of soft, clean, dry bedding or lying mats not only prevents leg injuries but also affects the length of resting time and the overall health of the cows.

However, it is important to note that due to changes in the surface profile, physical modifications can increase the likelihood of slips and injuries. In addition, elements such as rubber chains or nylon nets can deteriorate, and the fragments can be ingested by animals.

### 5.2. Socialisation

Another key consideration in cattle welfare is ensuring social contact (Table 2). On most farms, calves are separated from their mothers within 24 h of birth, which, although stressful, is associated with less intense behavioural responses compared to later separation, e.g., after two weeks [92,93]. Separation causes an increase in motor activity and more frequent cries (approximately four times) by mother and offspring, indicating high levels of stress [92,94].

The need for social contact in calves appears as early as the first week of life [95]. Research by Holm et al. [96] indicates that calves strongly prefer full physical contact with peers over limited contact through bars. Furthermore, full social contact from birth promotes the formation of stronger group bonds [97], and the bonds established early on appear to influence social preferences in adulthood [98,99,100,101].

Reduced social contact has negative effects on calf welfare. Isolation in the first months of life can result in increased timidity, adaptation problems, and impaired cognitive abilities in adulthood [102,103,104,105]. For example, eight-month isolation had a negative effect on the social behaviour of calves, who at 20 months of age were more likely to choose solitude and were found to rank lower in the group hierarchy [106].

However, socialising new individuals in a group of cattle can also result in increased stress and aggressive behaviour. Increased animal density also promotes the transmission of pathogens and can impair the health of the herd, especially in cases where sanitary conditions may be inadequate.

### 5.3. Nutrition

Another important part of animal welfare is adequate nutrition. Consumption of colostrum by calves not only promotes their growth and weight gain but also supports the maturation of the digestive tract [107]. Satisfying the need to suckle is very important to prevent undesirable behaviour such as mutual suckling of body parts such as ears, navel, or tail. Mutual suckling between calves (cross-suckling) merits attention from the farmer, as it may signal health and production problems in the future. The first cause of cross-suckling may be neglect of the natural sucking reflex. Many commercially produced teats are too soft or lose their hardness after prolonged use. If the calf can access the liquid feed very easily, it can result in faster weaning, which is not good for the animal. To ensure proper weaning, the calf needs to make two basic movements: pressure on the teat (pressure) and suction (vacuum). If only one movement is required to take the liquid feed, the calf does not satisfy its natural sucking reflex, leading to cross-suckling of other calves [108].

Cross-suckling can also be caused by the calf’s body being in an abnormal position when drinking: when the calf does not lift its head and make the characteristic sucking movement, this can disrupt the gutter reflex, causing some of the undigested feed to end up in the rumen, resulting in fermentation, rumen acidification, flatulence, and abdominal pain. In addition, low pH in the rumen leads to an increase in suckling activity in calves as they try to stimulate the production of saliva as a natural buffer. A similar effect occurs when the teat allows feed to be taken up too quickly, leading to overflow of feed over the gutter folds into the rumen. Both cases result in a higher frequency of cross-suckling [109,110].

Therefore, to prevent cross-suckling, which can adversely affect the development of the udder in calves and increase the risk of mastitis in the future, it is important to use high-quality teats and to provide the calves with an appropriate body position during weaning. Also to effectively meet their natural needs and reduce the frequency of suckling, calves should be provided with artificial teats, e.g., in the form of a milk bucket or pen fittings [109,110]; a dummy can also be provided for a longer period of time after feeding [111]. However, the use of poorly designed teats can lead to abnormal sucking patterns in calves as they may not satisfy the physiological need to suckle. In addition, an abnormal drinking position or excessive feed intake can cause metabolic problems due to feed entering the rumen.

Enrichment of the feeding environment can also lead to abnormal sucking patterns in calves, especially if poorly designed teats are used. Furthermore, where suckling is made too easy, the process may not satisfy the physiological need to suckle; this can lead to mutual suckling, which can result in udder damage in calves and an increased risk of mastitis. In addition, an incorrect drinking position or excessively rapid feeding can cause metabolic problems due to food entering the rumen.

### 5.4. Brushes and Stimulating Elements

The installation of cow brushes, rubber chains, or nylon nets filled with hay stimulates activity among calves, thus improving their mental and physical development. The elements encourage exploration and play, and calves have been found to spend several minutes a day at the brushes. Such activity has been found to improve the mood of the calves, reduce the effects of stress, and enhance their well-being and is believed to satisfy their natural need for grooming, which is often carried out in the wild by their mothers [112,113]. Furthermore, Pempek et al. [114] report that calves prefer brushes over other available objects (*p* < 0.05) such as a rubber chain or an artificial teat. Generally speaking, calves kept in groups with access to toys are less likely to exhibit undesirable behaviours, such as sucking on pen parts [114,115].

Adding brushes and toys such as rubber chains or nylon nets to the environment also poses risks, as such items can break and their fragments can be swallowed by the animals.

Enriching the environment of cattle is a complex process that involves physical, social, and nutritional modifications. These activities not only improve the comfort and health of animals but can also reduce the chance of undesirable behaviour.

### 5.5. Enriching the Environment and Psychological Stimulation

Recent research indicates that an effective housing system should aim to not only reduce stress in animals but also actively foster positive emotions. This should be achieved by providing a suitably-designed environment that enables the animals to perform their natural behaviours and that stimulates the neurobiological systems responsible for experiencing pleasure (Table 2) [116,117,118].

Mechanical brushes (tactile stimulation) play an important role in modern cattle environmental enrichment strategies. Cows are highly motivated to use these devices, sometimes even preferring them to access to fresh feed [119]. The use of brushes significantly increases the grooming time by 508% and the scratching frequency by 226% and reduces inactivity periods and aggression in the herd [120,121,122,123].

Sonic enrichment is also becoming increasingly important. Classical music can reduce stress in cows and increase milk production. It is best to use slow or moderate tempos (70–100 beats per minute) and keep the volume below 70 dB [117].

In addition, visual stimuli can play an important role in improving animal welfare, especially in intensive production systems. Such approaches are more effective when integrated with other forms of environmental enrichment, such as sound or olfactory stimuli, creating a multi-sensory and more natural environment for cattle. One practical solution for supporting animal welfare during short-term isolation (e.g., weighing, veterinary procedures) is the use of mirrors, which can alleviate the stress of loneliness by substituting for the presence of other individuals.

Piller et al. [124] report that the mean heart rate of heifers living in an environment enriched with mirrors was more than six beats per minute lower than that recorded for control animals. Significantly lower motor activity was also observed than in the controls, i.e., 34.8 MMD peaks per day vs. 68.9 MMD day^−1^, which indicates reduced stress and greater calmness in the mirror group. However, Mandel et al. [125] note only minimal differences in heart rate between groups. These results indicate that the mere presence of an image in a mirror may not be sufficient to induce a calming effect, especially in the absence of accompanying stimuli, such as movement or smell.

Pictures of familiar cows have proven to be more effective than mirrors. Ninomiya and Sato [85,126] found that showing a cow a natural-sized picture of the same breed reduced the level of cortisol in saliva by as much as 70% (from 0.33 to 0.22 pmol/mL) compared to cows presented with a blank wall. In addition, a significant reduction in vocalisation was observed (from 3.7 to 0). The effect on reducing stress was weakened if the cow was shown a picture of a stranger. However, the strongest calming effect was obtained in the actual presence of another cow, where the cortisol level was also 0.22 pmol/mL, and vocalisation was completely eliminated.

Although neither mirrors nor photos can fully replace natural social contact, due to the lack of movement, smell, and interaction between the animal and its environment, they can support stress reduction in certain situations.

A new approach to studying boredom in dairy cows housed in confined conditions was introduced by Russell et al. [127], who focussed on behavioural indicators of boredom and the impact of simple forms of environmental enrichment. The authors distinguished two new types of boredom behaviours. The first, idling or aimless standing, was 19% less common after the introduction of toys. The second, refusals or refusals to milk by robots, decreased by 0.5 per day after the introduction of environmental enrichment. The addition of enrichment, i.e., the placement of an inflatable buoy in the resting area, resulted in a mean period of 12 min of play per day in the first week and a reduction in idling and refusals. In addition, an increase in self-grooming was noted in the toy area (64% vs. 29% in the control group), which the authors interpret as a sign of positive arousal, although they note that self-grooming has been associated with stress in other studies; this aspect requires further investigation. The study concludes that boredom is a serious problem in cows in pastureless systems, and that it can be reduced even with simple environmental enrichment. Furthermore, monitoring behaviours such as idling and refusals can be a useful tool for assessing animal welfare. It can be seen that these new indicators require further exploration.

## 6. Conclusions

No matter which housing system is used, the welfare of cattle depends on the interaction of a number of factors, such as social bonds, access to space, breed, herd structure, and interactions with humans. The analysis clearly shows that keeping the husbandry conditions as close as possible to the natural environment enhances well-being amongst cattle. Creating conditions that foster lasting relationships, and satisfying the basic behavioural needs, rest, and freedom of movement result in significantly better health and well-being of animals.

Intensive systems, although characterised by high production efficiency, are often associated with increased stress, higher incidence of disease, and reduced natural behaviour, which negatively affects their welfare. In contrast, extensive systems, although less productive, favour the maintenance of natural behaviour and better animal welfare.

By providing greater freedom of movement and opportunities for social interaction, the freestall system promotes better mental and physical well-being among the animals: it has been found to reduce stress and promote natural behaviours such as moving and resting. However, careful management is needed to minimise the risk of limb disease and herd conflict. In contrast, a tethering system provides better control of the animals and reduces the risk of competition for resources, but it often restricts freedom of movement and thus negatively affects the welfare and health of the cows.

When improving the welfare of cattle, it is particularly important to ensure appropriate maintenance conditions and meet their nutritional needs. This will directly translate into improved animal health and increased productivity as well as reduced suffering and stress.

## Figures and Tables

**Table 1:** The effects different cattle housing systems on animal behaviour and their relationships.

Aspect	Free-Living Cattle	Beef Cattle Extensive System	Beef Cattle Intensive System	Dairy Cattle
The mother–calf bond	Close bond between mothers and adult daughters	Good bond between mothers and adult daughters	Mothers and daughters often separated	Mothers and daughters often separated
Social bonds of calves	Close social bonds between calves	Close social bonds between calves	Calves often raised in technological groups	Calves often raised individually
Age structure in the herd	Complex age structure in herds with long-term ties between individual animals	Complex age structure in flocks	New animals frequently introduced into the herd.	Selection and new animals frequently introduced into the herd.
Behaviour of mother and calf	Cows and calves always stay together	Cows and calves stay together	Cows and calves are often housed separately	Usually, calves are separated from their mothers immediately after birth
Access to pasture	Permanent access to pasture	Permanent access to pasture or from spring to late autumn	Often no access to pasture	Often no access to pasture
Hierarchy in the herd	Herd, hierarchy	Herds, looser structure	Groups, limited interaction between individuals	Groups, segregation
Space and natural behaviour	Free movement, ability to express natural behaviour	Sufficient space for basic natural behaviour	Limited space, limited possibility to express natural behaviour	Limited space, limited possibilities to express natural behaviour
Social interaction	Social interaction frequent, varied	Social interactions frequent but less diverse	Social interaction limited	Social interaction limited
Disease resistance	Good immunity, diseases rare	Lower immunity, parasitic diseases	Increased susceptibility to disease	Frequent metabolic diseases and mastitis
Stereotypic behaviour	Stereotypic behaviour rare	Stereotypic behaviour rare	Stereotypic behaviour frequent	Stereotypic behaviour frequent
Access to rest areas	Ample space for free movement	More space, but more restricted than wild cattle	Limited space, often restricted to barns or small paddocks	Limited space, often restricted to barns or small paddocks
Stress level	Stress level low, no human intervention	Stress level low but dependent on conditions (e.g., feed, space)	Stress level high, caused by barn conditions	Medium to high stress level, dependent on conditions and milking method
Staffing	little	little	a lot	a lot
Rest	Long periods of rest in natural conditions	Rest during the day, but conditions may be limited	Limited access to resting space, often uncomfortable conditions	Limited access to resting space, often uncomfortable conditions
Social interaction with humans	Lack of human interference in social interactions, natural social structures	Sufficient interaction with other animals, low human interference	High human intervention, frequent human interaction	Interaction with humans related to milking and handling
Environmental conditions	Natural environmental conditions, adequate temperature and ventilation	Under natural conditions, access to cover in winter	High risk of inadequate ventilation and temperature in intense conditions	High risk of inadequate ventilation and temperature in intense conditions

**Table 2:** Forms of environmental enrichment.

Form of Enrichment	Description	Example	Aim
Social	Interaction with other individuals or species	Contact with other individuals of the same species, interaction with keepers, observation of other animals	Stress reduction, stimulation of social behaviour, development of communication skills
Physical	Modifications to the physical environment	Changing the size of the enclosure, adding various objects to the barn (e.g., chiropody, rubber chain, artificial teats)	Stimulating physical activity, providing opportunities to explore the environment
Nutrition	Feed manipulation	Hiding food, feeding in different containers (e.g., hay net), play feeding	Stimulating the senses, encouraging active foraging
Activity	Mentally stimulating tasks		Development of cognitive abilities, reduction in boredom
Sensorial	Stimulation of the senses	Sounds, smells, variety of materials	Visual, audio, olfactory, and tactile stimulation

## Data Availability

Not applicable.
